# Donor Age and Non-Relapse Mortality: Study of Their Association after HLA-Matched Allogeneic Hematopoietic Cell Transplantation for Acute Myeloid Leukemia and Myelodysplastic Syndrome

**DOI:** 10.3390/curroncol29080470

**Published:** 2022-08-22

**Authors:** Yasmine Kadri, Michelle Phan, Nadia Bambace, Léa Bernard, Sandra Cohen, Jean-Sébastien Delisle, Thomas Kiss, Sylvie Lachance, Denis-Claude Roy, Guy Sauvageau, Olivier Veilleux, Jean Roy, Imran Ahmad

**Affiliations:** 1 Département de Médecine, Faculté de Médecine, Université de Montréal, Montreal, QC H3T 1J4, Canada; 2 Département de Pharmacologie & Physiologie, Faculté de Médecine, Université de Montréal, Montreal, QC H3T 1J4, Canada; 3 Institut Universitaire d’Hématologie-Oncologie & Thérapie Cellulaire de Montréal, Hôpital Maisonneuve-Rosemont, Montreal, QC H1T 2M4, Canada

**Keywords:** acute myeloid leukemia, myelodysplastic syndrome, allogenic hematopoietic cell transplantation, donor age, non-relapse mortality, graft-versus-host disease

## Abstract

The purpose of this retrospective study was to study the correlation between donor age (DA) and non-relapse mortality (NRM) and relapse incidence (RI) among patients treated with allogeneic hematopoietic cell transplantation (aHCT) for acute myeloid leukemia (AML) or myelodysplastic syndrome (MDS) in a single Canadian center. Data from 125 consecutive patients transplanted with a matched related or unrelated donor between 2015 and 2020 were analyzed using multivariable models. After a median follow-up of 2.8 years, the cumulative incidences of NRM and relapse were 19% and 35% at 5 years. Despite being independently associated with NRM and relapse-free survival (RFS), DA was not associated with RI. The independent determinants of NRM in addition to DA were patient age and hematopoietic cell transplantation comorbidity index (HCT-CI), independently of donor kinship. The effect of DA on NRM was found to be significantly increased over the age of 50 years. DA was not associated with incidence of acute graft-versus-host disease (aGVHD) but showed an association with the occurrence of chronic GVHD (cGVHD). In conclusion, younger donors should be favored to limit NRM and increase RFS in HLA-matched aHCT. The etiological mechanisms behind the association of DA with higher NRM remain to be elucidated.

## 1. Introduction

Allogeneic hematopoietic cell transplantation (aHCT) is the only curative treatment for most myeloid hematological malignancies [1]. In addition to the risk of malignancy relapse, many factors compromise the full curative potential of aHCT, such as drug-induced organ toxicity, infections and graft-versus-host disease (GVHD) [1,2]. Thus, determining the clinical variables leading to such complications may help predict the outcome for patients undergoing aHCT.

The median age of adult patients diagnosed with acute myeloid leukemia (AML) and myelodysplastic syndrome (MDS) is 65 and 75 years old, respectively, with both diseases being the main indications of aHCT in adults [3]. With the reduction in aHCT-related toxicity [4], the procedure has become accessible to older patients and those with comorbidities [1,3,5]. In clinical practice, an HLA-matched sibling donor remains the first option [6], leading to a progressive increase in the age of HLA-matched related donors, as their siblings are of similar age.

In past studies, younger donor age (DA) has generally been correlated with beneficial outcomes, but its differential effect on survival, relapse, non-relapse mortality (NRM) and acute and chronic GVHD (aGVHD and cGVHD) remains unclear [7,8,9,10,11,12]. Furthermore, most studies looking at the impact of DA on the outcomes have included various transplant indications, conditioning regimens, GVHD prophylaxis and pooled data from different centers.

The aim of this study was to determine the association between DA and the outcomes of aHCT for AML and MDS among patients treated according to similar procedures in a single center. Our hypothesis was that younger donors are associated with better outcomes following aHCT with a lower incidence of NRM and GVHD.

## 2. Materials and Methods

In this retrospective observational cohort, we included all consecutive adult subjects who underwent their first HLA-matched (8/8) aHCT between 1 July 2015 and 30 June 2020 at our institution. The exclusion criteria were: aHCT from a haploidentical donor, single or double cord blood unit transplants or an experimentally manipulated graft. Data were collected from electronic medical records after approval from the Research Ethics Committee.

The primary outcome was the cumulative incidence (CI) of NRM according to DA. The secondary outcomes included cumulative incidences of relapse, aGVHD, cGVHD and relapse-free survival.

### Statistical Analysis

The Kaplan–Meier method was used for the estimation of overall and relapse-free survival. The outcomes were timing from aHCT to relapse (or progression, if disease was not in remission at aHCT), grade II to IV aGVHD, moderate & severe or severe grade cGVHD and death. We estimated the cumulative incidences of relapse, NRM and GVHD using the competing-risk cumulative function. Death was considered as a competing risk for the estimation of cumulative incidences, except if occurring after 6 months for aGVHD and 2 years for cGVHD. Multivariate analyses using the Cox proportional hazards model and Fine–Gray model for cumulative incidences were used to study the association of pre-aHCT characteristics, including DA, with outcomes. DA, patient-age-adjusted HCT-comorbidity index (aaHCT-CI), Karnofsky performance status (KPS), donor type (matched related (MRD) or unrelated (MUD)), conditioning regimen intensity, sex mismatch (female donor to male recipient versus other), recipient CMV status, disease relapse risk (using the EBMT Disease Risk Stratification System), anti-thymocyte globulin (ATG) use and cell source (peripheral blood or bone marrow grafts) [13] were used as covariates when appropriate for the study outcomes. DA groups using decades were used to better define the association between DA and NRM.

## 3. Results

### 3.1. Baseline Characteristics

The studied cohort described in Table 1 included 125 patients. The median follow-up was 2.8 years (95% confidence interval (95% CI): 2.4–3.3). The median recipient age was 56 years (range: 18–70). DA ranged from 18 to 74 years, with a median age of 32 years old, with most MUDs under 50 and most MRDs over 50 years of age. Peripheral blood was the major source of hematopoietic cells, and MUD was the most common type of donor (67%). The conditioning regimens were myeloablative for 90% of the studied population, and most of the GVHD prophylaxis regimens included a calcineurin inhibitor (either ciclosporin or tacrolimus) combined with methotrexate (82%) or mycophenolate mofetil (18%). Rabbit ATG was included in the GVHD prophylaxis in almost all MUD peripheral blood aHCT.

### 3.2. Survival and Relapse

**Figure 1:**
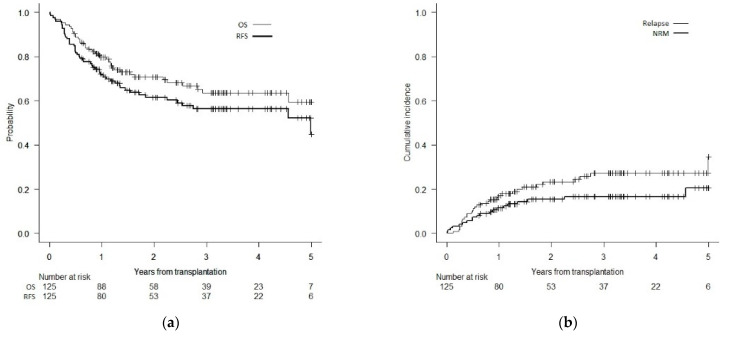
(**a**) Overall and relapse-free survival. (**b**) Cumulative incidences of relapse and non-relapse mortality.

The overall survival and RFS at 5 years were 59% (95% CI: 46–70) and 45% (95% CI: 28–61), respectively (Figure 1a). The cumulative incidences of relapse and NRM at 5 years were 35% (95% CI: 20–50) and 21% (95% CI: 12–31), respectively (Figure 1b). DA was not associated with OS, the incidence of relapse or RFS in the multivariate analysis.

### 3.3. Association between Donor Age and Non-Relapse Mortality

The multivariable analysis for NRM as the dependent variable included recipient age, HCT-CI, KPS, conditioning intensity, CMV serostatus, donor type, disease risk score and sex mismatch. After model selection using the Bayesian information criterion, the only variables showing a significant and independent association with NRM were recipient age, HCT-CI (HR for aaHCT-CI: 1.90; *p* = 0.04) and DA (HR for DA as continuous variable: 1.04; *p* = 0.03).

**Figure 2:**
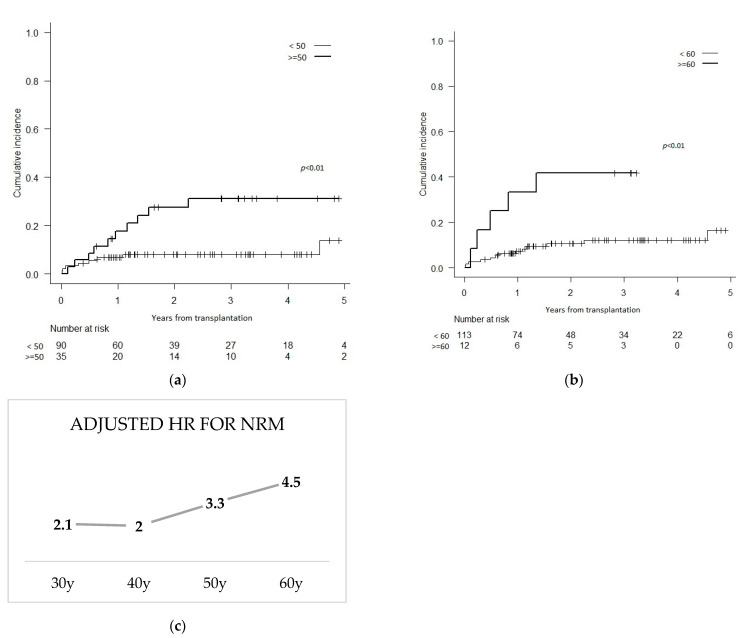
Cumulative incidence of NRM among patients with donor aged (**a**) <50 years versus ≥50 years; (**b**) <60 years versus ≥60 years; (**c**) adjusted hazard ratios (HR) of various donor ages for the association with NRM (reference is <30 y).

We then divided the patients into decade-based age categories (i.e., <30, 30–39, 40–49], 50–59 and ≥60) and found a strong but statistically non-significant association between DA and NRM at age ≥40 (HR 2.03; *p* = 0.14). This association became statistically significant at a DA ≥ 50 years (HR 3.35; *p* = 0.01) and even stronger at 60 years and older (HR 4.54; *p* = 0.01) (Figure 2). This association between the DA and NRM is reflected in the PFS.

### 3.4. Association between Donor Age, Graft-versus-Host Disease and Non-Relapse Mortality

To test the hypothesis that older DA causes more GVHD, which in turn could explain a higher NRM, we developed multivariable models with aGVHD grades II–IV, III–IV and cGVHD grades of moderate–severe and severe with known and suspected risk factors, including DA. There was no statistically independent and significant association between aGVHD and DA, but the model selection yielded two independent and significant variables associated with the occurrence of moderate–severe and severe cGVHD: previous aGVHD (for moderate–severe: HR 5.6, *p* < 0.001, for severe: HR 5.2, *p* < 0.001) and DA (HR with DA as a continuous variable for moderate–severe: HR 1.03, *p* = 0.017, for severe: HR 1.03, *p* = 0.032). Unexpectedly, ATG use was not significantly associated with cGVHD (*p* = 0.41).

## 4. Discussion

Since the 1980s, the beneficial effects of a younger donor on aHCT outcomes have been suggested to be a lower risk of acute GVHD and even a lower risk of relapse in allogeneic bone marrow recipients [14,15]. The association of an older donor with increased GVHD and overall mortality was confirmed in a large cohort of nearly 7000 recipients of MUD marrow grafts [9]. Later studies, mostly reporting data with peripheral blood stem cell transplants, reported either no association of DA with transplant outcomes or even better outcomes with older donors (MRD, mostly), with recommendations not to take donor age into account for aHCT in elderly patients with AML [7,11,16,17,18]. In the more recently published literature, younger DA was again being proven to be associated with better survival due to lower incidences of GVHD and NRM [10,12,19,20]. The different results looking at the relationship between DA and the outcomes of aHCT may be explained by changes in the graft source over time (marrow to peripheral blood grafts), different GVHD prophylaxis (e.g., in vivo T-cell depletion being more common in recent years) and better donor selection (high-resolution HLA typing for MUD).

Our cohort represents modern aHCT with high-resolution HLA-typing donor selection, where most patients received ATG, particularly in peripheral blood stem cells transplantation. With these conditions, DA remains associated with NRM, and to some extent, with cGVHD. The higher mortality rate with older donors was initially attributed to the increased incidence of GVHD and related complications [21], but the mechanism behind better survival with younger donors was associated neither with lower NRM nor lower GVHD [8]. This observation is in line with recent reports with modern haploidentical aHCT using immunomodulation with post-transplant cyclophosphamide [22,23,24,25].

In our study, we observed higher incidences of NRM and cGVHD with older DA, but cGVHD was not associated with a significant increase in NRM. It is indeed known that the chronic form of GVHD does not carry a large burden of mortality. We therefore hypothesize that other factors, such as infectious complications, dysimmunity and organ toxicities (not mediated by GVHD), can explain the higher NRM observed with older DA. Infectious complications are often not detailed in registry data and are therefore more difficult to assess in large cohorts. Organ failures can be a consequence of infections, even months and years after aHCT, and this hypothesis is supported by studies showing lower immune reconstitution markers (lymphocyte populations, particularly CD4^+^) in aHCT recipients of older donors [26,27,28].

The optimal cut-off for DA remains unknown, but worse survival has been shown with donors over the age of 35 or 45 years old [29,30]. More recent data showed a continuous relationship between aging DA and lower survival [19], which is concordant with our observations. Our data show a linearly increasing risk of NRM according to DA, reaching statistical significance over the age of 50. This age limit may, however, be influenced by the relatively small sample size, as the point estimate of HRs starts increasing from the age of 40.

As to whether a younger MUD should be preferred over an older MRD, the question remains open until a prospective trial is available. Nevertheless, recent data show similar overall survival after MUD and MRD aHCT, although sometimes with less NRM but increased relapse rates in MRD [31]. We believe that recent efforts to minimize GVHD, as the use of ATG serum and post-transplant cyclophosphamide, will help overcome the higher NRM associated with MUD transplants. In our cohort, older donors were predominantly MRDs, and only 10% of those transplants included ATG, whereas 49% of MUD procedures included ATG. Notwithstanding the inclusion of donor type and ATG use, the multivariable analyses revealed the independent effect of DA on NRM and cGVHD. However, a certain level of collinearity cannot be overcome even with multivariable analyses. Our cohort, even treated in a single center, using uniform protocols, suffers from heterogeneity because of changes in practice over time. Therefore, future studies using uniform in vivo T-cell depletion are required to assess the independent effect of DA on GVHD and NRM.

Our study has limitations inherent to its retrospective nature, but all previous observations on DA and aHCT outcomes were also retrospective. Although we restricted our study to the most frequent indications of aHCT in adults and used multivariable analyses, we included a heterogenous group of donor types, cell sources, conditioning and GVHD prophylaxis regimens. The absence of infection data in our cohort is a limitation of our study.

## 5. Conclusions

In conclusion, understanding the factors impacting NRM remains challenging and an impediment to achieving the full curative potential of aHCT. Our retrospective study shows that in contemporary HLA-matched transplants, choosing a younger donor may help reduce NRM. An optimal age cut-off for donor selection cannot be determined, as the association of DA with NRM is continuous. The mechanisms behind worse outcomes with older donors are not fully understood and are not solely mediated by GVHD. These observations should be tested in prospective trials comparing MRDs to younger MUDs with several aHCT indications and studying the impact of the waiting time to find the best, youngest MUD when choosing over an MRD.

## Figures and Tables

**Table 1:** Population characteristics.

**Total Number of Subjects**	**125 (%)**
Recipient age, years (median/range/IQR)	56/18–70/50–62
Donor age, years (median/range/IQR)	32/18–74/24–52
Donor type	
MRD (8/8)	41 (33)
MUD (8/8)	84 (67)
Donor age (years)/type	
<50 MRD	10 (8)
<50 MUD	80 (64)
≥50 MRD	31 (25)
≥50 MUD	4 (3)
Disease	
Acute Myeloid Leukemia	89 (71)
Myelodysplastic syndrome	36 (29)
Disease relapse risk (DRSS)	
Low	23 (19)
Intermediate-1	72 (58)
Intermediate-2	16 (13)
High	9 (7)
Very high	4 (3)
Recipient Karnofsky Performance Status	
<90%	47 (38)
≥90%	77 (62)
Recipient HCT-CI	
0	37 (30)
1–2	46 (37)
≥3	40 (32)
Cell source	
Bone marrow	33 (26)
TNC range (×10^8^/kg)	1.20–6.51
Peripheral blood	92 (74)
CD34^+^ cell range (×10^6^/kg)	2.44–14.45
CMV serostatus positive (recipient)	46 (37)
Sex mismatch (female donor to male recipient)	22 (18)
Conditioning regimen intensity	
Myeloablative	112 (90)
Reduced intensity	13 (10)
GVHD prophylaxis	
MTX/CnI	103 (82)
MMF/CnI	22 (18)
rATG *	46 (37)

Abbreviations. CMV: cytomegalovirus, CnI: calcineurin inhibitor, DRSS: Disease Risk Stratification System, HCT-CI: hematopoietic cell transplantation comorbidity index, IQR: interquartile range, MMF: mycophenolate mofetil, MTX: methotrexate, MRD: matched related donor, MUD: matched unrelated donor, rATG: rabbit anti-thymocyte globulin, TNC: total nucleated cell. * Standard for peripheral blood MUD aHCT before 2015, introduced as standard for MRDs in 2019.

## Data Availability

The dataset will be provided on demand.

## References

[1] D’Souza A., Fretham C., Lee S.J., Arora M., Brunner J., Chhabra S., Devine S., Eapen M., Hamadani M., Hari P. (2020). Current Use of and Trends in Hematopoietic Cell Transplantation in the United States. Biol. Blood Marrow Transpl..

[2] McDonald G.B., Sandmaier B.M., Mielcarek M., Sorror M., Pergam S.A., Cheng G.S., Hingorani S., Boeckh M., Flowers M.D., Lee S.J. (2020). Survival, Nonrelapse Mortality, and Relapse-Related Mortality after Allogeneic Hematopoietic Cell Transplantation: Comparing 2003–2007 versus 2013–2017 Cohorts. Ann. Intern. Med..

[3] Passweg J.R., Baldomero H., Chabannon C., Basak G.W., de la Cámara R., Corbacioglu S., Dolstra H., Duarte R., Glass B., Greco R. (2021). Hematopoietic cell transplantation and cellular therapy survey of the EBMT: Monitoring of activities and trends over 30 years. Bone Marrow Transpl..

[4] Penack O., Peczynski C., Mohty M., Yakoub-Agha I., Styczynski J., Montoto S., Duarte R.F., Kröger N., Schoemans H., Koenecke C. (2020). How much has allogeneic stem cell transplant-related mortality improved since the 1980s? A retrospective analysis from the EBMT. Blood Adv..

[5] Lin R.J., Artz A.S. (2021). Allogeneic hematopoietic cell transplantation for older patients. Hemat. Am. Soc. Hematol. Educ. Program.

[6] Shouval R., Fein J.A., Labopin M., Kröger N., Duarte R.F., Bader P., Chabannon C., Kuball J., Basak G.W., Dufour C. (2019). Outcomes of allogeneic haematopoietic stem cell transplantation from HLA-matched and alternative donors: A European Society for Blood and Marrow Transplantation registry retrospective analysis. Lancet Haematol..

[7] Alousi A.M., Le-Rademacher J., Saliba R.M., Appelbaum F.R., Artz A., Benjamin J., Devine S.M., Kan F., Laughlin M.J., Lazarus H.M. (2013). Who is the better donor for older hematopoietic transplant recipients: An older-aged sibling or a young, matched unrelated volunteer?. Blood.

[8] Kröger N., Zabelina T., de Wreede L., Berger J., Alchalby H., van Biezen A., Milpied N., Volin L., Mohty M., Leblond V. (2013). Allogeneic stem cell transplantation for older advanced MDS patients: Improved survival with young unrelated donor in comparison with HLA-identical siblings. Leukemia.

[9] Kollman C., Howe C.W., Anasetti C., Antin J.H., Davies S.M., Filipovich A.H., Hegland J., Kamani N., Kernan N.A., King R. (2001). Donor characteristics as risk factors in recipients after transplantation of bone marrow from unrelated donors: The effect of donor age. Blood.

[10] Kollman C., Spellman S.R., Zhang M.J., Hassebroek A., Anasetti C., Antin J.H., Champlin R.E., Confer D.L., DiPersio J.F., Fernandez-Viña M. (2016). The effect of donor characteristics on survival after unrelated donor transplantation for hematologic malignancy. Blood.

[11] Rezvani A.R., Storer B.E., Guthrie K.A., Schoch H.G., Maloney D.G., Sandmaier B.M., Storb R. (2015). Impact of donor age on outcome after allogeneic hematopoietic cell transplantation. Biol. Blood Marrow Transpl..

[12] Bastida J.M., Cabrero M., Lopez-Godino O., Lopez-Parra M., Sanchez-Guijo F., Lopez-Corral L., Vazquez L., Caballero D., Del Cañizo C. (2015). Influence of donor age in allogeneic stem cell transplant outcome in acute myeloid leukemia and myelodisplastic syndrome. Leuk. Res..

[13] Shouval R., Fein J.A., Labopin M., Cho C., Bazarbachi A., Baron F., Bug G., Ciceri F., Corbacioglu S., Galimard J.E. (2021). Development and validation of a disease risk stratification system for patients with haematological malignancies: A retrospective cohort study of the European Society for Blood and Marrow Transplantation registry. Lancet Haematol..

[14] Atkinson K., Farrell C., Chapman G., Downs K., Penny R., Biggs J. (1986). Female marrow donors increase the risk of acute graft-versus-host disease: Effect of donor age and parity and analysis of cell subpopulations in the donor marrow inoculum. Br. J. Haematol..

[15] Jacobsen N., Badsberg J.H., Lönnqvist B., Ringdén O., Volin L., Rajantie J., Nikoskelainen J., Keiding N. (1990). Graft-versus-leukaemia activity associated with CMV-seropositive donor, post-transplant CMV infection, young donor age and chronic graft-versus-host disease in bone marrow allograft recipients. The Nordic Bone Marrow Transplantation Group. Bone Marrow Transpl..

[16] Richa E.M., Kunnavakkam R., Godley L.A., Kline J., Odenike O., Larson R.A., Nguyen V., Stock W., Wickrema A., Van Besien K. (2012). Influence of related donor age on outcomes after peripheral blood stem cell transplantation. Cytotherapy.

[17] Segal E., Martens M., Wang H.L., Brazauskas R., Weisdorf D., Sandmaier B.M., Khoury H.J., de Lima M., Saber W. (2017). Comparing outcomes of matched related donor and matched unrelated donor hematopoietic cell transplants in adults with B-Cell acute lymphoblastic leukemia. Cancer.

[18] Sorror M.L., Storb R.F., Sandmaier B.M., Maziarz R.T., Pulsipher M.A., Maris M.B., Bhatia S., Ostronoff F., Deeg H.J., Syrjala K.L. (2014). Comorbidity-age index: A clinical measure of biologic age before allogeneic hematopoietic cell transplantation. J. Clin. Oncol..

[19] Shaw B.E., Logan B.R., Spellman S.R., Marsh S.G.E., Robinson J., Pidala J., Hurley C., Barker J., Maiers M., Dehn J. (2018). Development of an Unrelated Donor Selection Score Predictive of Survival after HCT: Donor Age Matters Most. Biol. Blood Marrow Transpl..

[20] Paz A., Rigoni L., Fischer G., Schittler M., Pezzi A., Valim V., Dahmer A., Zambonato B., Amorin B., Sehn F. (2018). Donor characteristics and hematopoietic stem cell transplantation outcome: Experience of a single center in Southern Brazil. Hematol. Transfus. Cell Ther..

[21] Flowers M.E., Inamoto Y., Carpenter P.A., Lee S.J., Kiem H.P., Petersdorf E.W., Pereira S.E., Nash R.A., Mielcarek M., Fero M.L. (2011). Comparative analysis of risk factors for acute graft-versus-host disease and for chronic graft-versus-host disease according to National Institutes of Health consensus criteria. Blood.

[22] Mariotti J., Raiola A.M., Evangelista A., Carella A.M., Martino M., Patriarca F., Risitano A., Bramanti S., Busca A., Giaccone L. (2020). Impact of donor age and kinship on clinical outcomes after T-cell-replete haploidentical transplantation with PT-Cy. Blood Adv..

[23] DeZern A.E., Franklin C., Tsai H.L., Imus P.H., Cooke K.R., Varadhan R., Jones R.J. (2021). Relationship of donor age and relationship to outcomes of haploidentical transplantation with posttransplant cyclophosphamide. Blood Adv..

[24] Karam E., Laporte J., Solomon S.R., Morris L.E., Zhang X., Holland H.K., Bashey A., Solh M.M. (2019). Who Is a Better Donor for Recipients of Allogeneic Hematopoietic Cell Transplantation: A Young HLA-Mismatched Haploidentical Relative or an Older Fully HLA-Matched Sibling or Unrelated Donor?. Biol. Blood Marrow Transpl..

[25] Ciurea S.O., Al Malki M.M., Kongtim P., Fuchs E.J., Luznik L., Huang X.J., Ciceri F., Locatelli F., Aversa F., Castagna L. (2020). The European Society for Blood and Marrow Transplantation (EBMT) consensus recommendations for donor selection in haploidentical hematopoietic cell transplantation. Bone Marrow Transpl..

[26] Baron F., Zachee P., Maertens J., Kerre T., Ory A., Seidel L., Graux C., Lewalle P., Van Gelder M., Theunissen K. (2015). Non-myeloablative allogeneic hematopoietic cell transplantation following fludarabine plus 2 Gy TBI or ATG plus 8 Gy TLI: A phase II randomized study from the Belgian Hematological Society. J. Hematol. Oncol..

[27] Azuma E., Hirayama M., Yamamoto H., Komada Y. (2002). The role of donor age in naive T-cell recovery following allogeneic hematopoietic stem cell transplantation: The younger the better. Leuk. Lymphoma.

[28] Hirayama M., Azuma E., Jiang Q., Kobayashi M., Iwamoto S., Kumamoto T., Kisenge R., Yamamoto H., Komada Y. (2000). The reconstitution of CD45RBhiCD4^+^ naive T cells is inversely correlated with donor age in murine allogeneic haematopoietic stem cell transplantation. Br. J. Haematol..

[29] Carreras E., Jiménez M., Gómez-García V., de la Cámara R., Martín C., Martínez F., Iriondo A., Sanz G., Cañizo C., Cabrera R. (2006). Donor age and degree of HLA matching have a major impact on the outcome of unrelated donor haematopoietic cell transplantation for chronic myeloid leukaemia. Bone Marrow Transpl..

[30] Mehta J., Gordon L.I., Tallman M.S., Winter J.N., Evens A.M., Frankfurt O., Williams S.F., Grinblatt D., Kaminer L., Meagher R. (2006). Does younger donor age affect the outcome of reduced-intensity allogeneic hematopoietic stem cell transplantation for hematologic malignancies beneficially?. Bone Marrow Transpl..

[31] Guru Murthy G.S., Kim S., Hu Z.H., Estrada-Merly N., Abid M.B., Aljurf M., Bacher U., Badawy S.M., Beitinjaneh A., Bredeson C. (2022). Relapse and Disease-Free Survival in Patients with Myelodysplastic Syndrome Undergoing Allogeneic Hematopoietic Cell Transplantation Using Older Matched Sibling Donors vs Younger Matched Unrelated Donors. JAMA Oncol..

